# Annual Review of Gerontology and Geriatrics Symposium: Black Older Adults in the Era of Black Lives Matter

**DOI:** 10.1093/geroni/igab046.181

**Published:** 2021-12-17

**Authors:** Jessica Kelley, Roland Thorpe, Linda Chatters

## Abstract

Our renewed urgency and engagement in a national dialogue on issues of systemic racism and racial justice provides a much-needed opportunity to expand the discourses, perspectives, and practices used in the study of aging. This symposium features contributions from the 2021 (Vol 41) Annual Review of Gerontology and Geriatrics focusing on the continued development and maturation of scholarship on the lives of older Black Americans. Building on the scholarship and research contributions of prior generations of eminent African American gerontologists, the volume asks: “What do we know about the lived experience of Black older adults and what is there still to be learned?” The contributing authors continue a tradition of research that examines the life histories and contemporary experiences of older Black adults within their relevant social and personal contexts. Symposium presenters from a range of social science fields (sociology, psychology, social work), explore aspects of physical health, stress, cognition, and social well-being in the context of intersecting social dimensions of marriage, family, gender, and neighborhood.

